# Strategies To Increase Alcohol Screening in Health Care Settings

**Published:** 1997

**Authors:** Michael F. Fleming

**Affiliations:** Michael F. Fleming, M.D., M.P.H., is professor of family medicine at the University of Wisconsin Medical School, Madison, Wisconsin

**Keywords:** identification and screening for AOD use, physician, education and training, professional education, questionnaire, clinic, outreach, group participation, medical screening and diagnostic method, job performance, evaluation, motivation, literature review

## Abstract

Although health care settings offer an ideal opportunity for identifying people who are currently experiencing or are at risk for problems with alcohol, clinicians screen fewer than one-half of their patients for alcohol use disorders. The rate of alcohol screening may be increased, however, by applying strategies shown to promote the use of screening procedures for other medical problems, such as cancer. These strategies include group education (e.g., workshops or seminars), training given by respected colleagues (i.e., opinion leaders), performance feedback, educational outreach visits to individual physicians (i.e., academic detailing), and financial incentives or penalties. Using clinic-based system protocols (e.g., patient questionnaires) can help make the implementation of alcohol screening in clinical practice both efficient and effective. Although incorporating alcohol screening into other high-priority clinical activities and screening programs remains a challenge, routine alcohol screening as a standard of care for all patients is receiving increased acceptance.

Both the U.S. Department of Agriculture and the Department of Health and Human Services recommend limiting alcohol consumption to no more than two standard drinks^1^ per day for men and no more than one standard drink per day for women and people over age 65. Alcohol use above these recommended limits is associated with a wide range of health-related concerns, including high blood pressure, trauma, accidents, domestic violence, cancer, fetal alcohol syndrome, and mental health problems. In fact, alcohol use disorders are some of the most common problems seen in health care settings. Studies suggest that 20 percent of the people who seek care in hospitals and outpatient clinics are at risk for or are experiencing alcohol-related problems (Fleming et al. 1998).

Because patients consider their doctors to be trusted and credible sources of health information, health care settings are ideal for implementing alcohol-screening procedures. Several screening tests to identify alcohol use disorders in patients have been developed for use in clinical settings. These tests are highly sensitive, specific, and similar in accuracy to a blood pressure measurement to detect high blood pressure or a glucose tolerance test to screen for diabetes. For patients who screen positive for alcohol use disorders, physicians can take action to promote healthy, successful outcomes. For example, both alcohol consumption and health care utilization decrease when clinicians incorporate simple procedures (i.e., brief interventions, such as providing written material and advice) into routine office visits with patients who are nondependent drinkers and provide specialized treatment for patients who are alcohol dependent (Fleming et al. 1997*a*).

## Prevalence of Alcohol Screening

Despite findings that support the implementation of routine alcohol screening and demonstrate its advantages, the rate of alcohol screening in health care settings remains lower than 50 percent, as several studies have noted. For example, Moore and colleagues (1989) conducted a survey in a large university hospital in Baltimore, Maryland, and found that physicians recorded an alcohol use history for only about one-third of their patients. Inpatient psychiatric units had the highest rates of screening in this study, and surgical units had the lowest. Another study conducted in the emergency department of a large teaching hospital surveyed 346 patients involved in motor vehicle crashes and found that physicians obtained the patient’s blood alcohol level in fewer than 25 percent of the cases (Chang and Astrachan 1988). One possible reason for such a low rate of alcohol screening may be related to medicolegal concerns. Clinicians may not realize that blood alcohol levels obtained in an acute care setting are not admissible as evidence for legal actions when the sampling does not follow a chain-of-custody collection procedure to safeguard it against any opportunity for interference (e.g., tampering).

Schmidt and colleagues (1995) found that 20 percent of the patients who participated in exit interviews at a general medical clinic reported that their physicians had asked them about their alcohol use in the previous 6 months. Of the 26 patients who were asked about their alcohol consumption, only 2 received specific recommendations. Patients who screened positive for a diagnosis of alcohol abuse or dependence (according to criteria set forth in the American Psychiatric Association’s *Diagnostic and Statistical Manual, Third Edition, Revised* [DSM–III–R]) were slightly more likely to have been asked about their alcohol use, but none of the patients who met current criteria for alcohol dependence were referred to a peer-support group, such as Alcoholics Anonymous, or to alcohol treatment. In another study, researchers assessed a sample of 972 adults in 2 rural primary care practices on their alcohol use (Fleming et al. 1995). Of the 110 patients who met DSM–III–R criteria for alcohol abuse or dependence, only 9 reported that their physicians had talked to them about their drinking in the last 6 months.

These studies suggest that physicians do not routinely screen their patients for alcohol use disorders. Too often, patients continue to be treated for alcohol-related trauma, high blood pressure, depression, anxiety, and other health problems without being treated for their underlying alcohol problem. Moreover, failing to screen for alcohol use disorders can result in serious clinical consequences. Surgeons and anesthesiologists, in particular, should consider alcohol screening as part of routine preoperative care, because alcoholics may require a greater amount of anesthesia to achieve the desired effect. In addition, delirium tremens may develop during the postoperative period, and alcohol withdrawal can severely compromise a patient’s recovery from surgical procedures. Similarly, patients admitted to trauma or coronary care units who develop delirium tremens are at greater risk for respiratory failure, blood flow restriction to the heart muscle (i.e., myocardial ischemia), and brain damage. Routine alcohol screening and early treatment of withdrawal will minimize the development of such complications.

## Strategies To Increase Alcohol Screening Rates

To increase alcohol screening rates in clinical settings, physicians must be encouraged to change their practice routines to include screening for every patient. Routine screening for all patients, however, involves overcoming barriers and issues in health care systems that currently block the way, such as the following:

- *Insufficient training in medical school and residency.*—Asking patients about sensitive lifestyle issues, such as their alcohol use, requires strong doctor-patient communication skills. Many physicians have not received skills training in this area.
- *Shortage of role models.*—Most physician groups have few role models with expertise in the alcohol field. However, difficult clinical areas, such as alcohol use disorders, require respected leaders to champion the cause and promote change.
- *Lack of performance feedback.*—Physicians do not routinely receive feedback on their performance regarding alcohol-related problems, because managed care companies and other health care systems have not made this area a priority.
- *Inadequate standards of care.*—Standard medical practice does not include obtaining an alcohol use history for every patient. In addition, quality assurance committees and accreditation groups have not penalized or cited physicians or health care institutions for having low rates of alcohol screening.
- *Inflexible clinic systems.*—Health care settings are complex systems that can be difficult to change. The introduction of a new clinical procedure, such as routine alcohol screening, requires a commitment by the entire clinic.

Several strategies have been found to be effective in promoting the use of screening procedures for other medical problems (e.g., cancer) in health care settings. These strategies can be classified into the following five general categories:

- Group education sessions
- Education by respected colleagues (i.e., opinion leaders)
- Performance feedback
- Educational outreach to individual physicians (i.e., academic detailing)
- Financial incentives or penalties.

Often, a combination of these types of strategies is used. Although their efficacy when applied to alcohol screening has not been widely tested, these strategies appear to offer a promising opportunity for the field. This article discusses each of the five types of strategies and presents reports of their effectiveness in other medical fields and in the alcohol field when available.

### Group Education Sessions

Courses, seminars, and workshops on screening practices and procedures are sometimes offered to groups of health care professionals. These educational opportunities are used to increase the rates of routine screening in clinical practice. Group education sessions can vary in their effectiveness, however, depending on their structure and content. Schwartz and Cohen (1990) describe education as the “provision of new information,” which is frequently necessary but not usually sufficient to change behavior. Physicians often require strong evidence before they will consider altering their routines. Therefore, change strategies that rely solely on providing new information without addressing the complex behavioral and organizational factors that influence physicians’ behavior are generally not successful.

Effective group education strategies include the following:

- Onsite educational programs at the physician’s clinic or hospital
- Specific, step-by-step, evidence-based, clinical protocols
- Peer group discussion
- Skills-based role-playing
- Credible experts delivering the training sessions.

Combining an educational program with some of the other intervention strategies presented later in this article (e.g., clinic-based system protocols and feedback from peers) also increases the program’s effectiveness (Davis et al. 1995). The National Institute on Alcohol Abuse and Alcoholism’s (NIAAA’s) development of a trainers’ manual for use with the Institute’s guide for physicians is one example of an educational program that incorporates many of these group education strategies (Fleming et al. 1997*b*).

Numerous researchers have examined the efficacy of group education in changing physician behavior and in its subsequent effects on improving patient health. Davis and colleagues (1995) surveyed the physician performance literature from 1975 to 1994 and found 160 studies that evaluated educational strategies, 99 of which were randomized clinical trials conducted in a variety of medical fields. Seventy percent of these studies reported changes in physician performance, and 48 percent of the studies that measured health outcomes found a positive change. The impact of the educational strategies varied among the different types of methods employed, however. Formal continuing medical education (CME) courses using lectures and handouts had limited impact, whereas educational programs that included peer discussion and skills-practice sessions were more effective.

A study by Dietrich and colleagues (1990) examined CME programs on controlling cancer and concluded that programs using interactive discussion groups, opinion leaders, and physician-formulated plans (i.e., protocols and procedures they selected for implementing a cancer screening program into their practice) result in improved knowledge and self-reported behavior change. Cohen and colleagues (1994) listed several factors associated with effective CME programs. For example, effectiveness was enhanced when the trainers were physicians identified by their peers as being respected and influential and when the trainers used multiple methods, especially methods that were designed not only to motivate physicians, but also to teach them new skills and help them change their practice environments.

Educational programs conducted for health care professionals on alcohol screening should incorporate the findings of all these reports. In particular, role playing can be an invaluable way to teach physicians how to become more comfortable with alcohol screening questions and interview techniques by allowing them to rehearse their skills before they interact with their patients. Because nothing can substitute for practice and repetition, role playing with colleagues, standardized patients (i.e., people trained to play a specific role), or people in recovery can build a physician’s confidence in his or her alcohol-screening skills. For example, role playing can help physicians learn to focus as much on what patients do not say (i.e., nonverbal cues) as what they do say when questioned about their alcohol use. Trainers can facilitate role playing in a small group, or if the group is large, trainers can use a paired role-play technique in which participants role play with the person sitting next to them.

### Opinion Leaders

Opinion leaders are respected colleagues who are trusted sources of clinical information. These leaders can be local physicians or colleagues known as experts at a State or national level. Often they are trained in the same specialty as the physicians to whom they are speaking.

The presentation of new information involving changes in clinical practice can be very effective when conducted by a trusted colleague. This effectiveness was demonstrated by a study done in the obstetrics field, in which the researchers performed a randomized, controlled trial with 76 physicians in 16 community hospitals to increase rates of vaginal births in women with previous histories of cesarean sections (Lomas et al. 1991). The trial included three groups of providers. First, a control group of physicians received a one-time mailing informing them of the recommended cesarean section guidelines and simply requesting that they implement these guidelines. A second group had their patients’ charts audited to compare actual practices with the recommended guidelines. This group of providers met quarterly for feedback and discussion on the audit results. The third group received written and oral communication from a physician nominated as an “educationally influential opinion leader,” who educated the physicians on the advantages and safety of vaginal births after a previous cesarean section. After 24 months, vaginal birth rates in the audit-and-feedback group were no different from those in the control group. The rates of cesarean section fell dramatically, however, among the physicians educated by an opinion leader. The patients of this group also had shorter hospital stays. No adverse clinical outcomes were attributable to any of the education efforts.

The use of respected colleagues as opinion leaders has special importance for the alcohol field, where societal and health care system barriers may impede the incorporation of alcohol screening into routine clinical care. Opinion leaders can help overcome these barriers by legitimizing and providing the scientific rationale for implementing alcohol-screening procedures. In addition, these leaders can counter societal biases and attitudes that place a lower value on spending health care resources for a so-called self-inflicted problem. Just as opinion leaders in the cardiology field can justify large expenditures to prevent and treat smoking-induced heart disease, opinion leaders in the alcohol field can provide the rationale for the prevention and treatment of alcohol use disorders.

One of the most promising developments in the alcohol field is the expanding number of faculty in primary care, obstetrics, emergency medicine, and surgery who are teaching their colleagues how to screen for alcohol problems. Opinion leaders such as these faculty members can play a major role in educating physicians and facilitating changes in physicians’ alcohol-screening practices. Although research on the effectiveness of using opinion leaders to change behavior is limited, this strategy appears to have potential.

### Performance Feedback

Changing a physician’s clinical behavior is not an easy process; however, providing feedback is one of the most powerful methods available, especially when a physician perceives a need for change in clinical care. According to Greco and Eisenberg (1993), feedback includes various ways of giving health care providers information about their practice performance and patient outcomes compared with the performance of other providers. Feedback can be used to introduce a new procedure, or it can be part of an overall clinic quality assurance system. Examples of effective feedback include confidential performance evaluations based on medical record reviews, written feedback by quality assurance committees, and feedback derived from patient satisfaction questionnaires. Peer-review feedback is increasingly used by managed care organizations to modify physician behavior, especially in the prevention field (e.g., to encourage immunizations and cancer prevention activities). Data gathered from peer-review feedback also are used to monitor the quality of care that patients receive as well as serve as the basis for financial incentives for physicians.

Researchers in various health fields have evaluated feedback as a tool for changing physician behavior. Through more than 30 years of research, Bowers and Franklin (1977) have shown that general organizational change can be greatly facilitated when data about systems functioning are collected, communicated to the organization’s members, and used to provide opportunities for diagnosis and action. Recent studies reviewed by Greco and Eisenberg (1993) demonstrate such changes in the health professions. Their research findings show the following results from feedback:

- Reductions in the length of hospital stays
- Reductions in the number of medications prescribed to outpatients
- Reductions in the number of outpatient tests ordered
- Increased compliance with guidelines (in this case, for cancer screening)
- Reductions in laboratory and total hospital costs.

Schwartz and Cohen (1990) describe some of the ways that feedback can be given. These include both impersonal means, such as providing computer profiles or reports, and personal interactions, such as through peer review groups or committees. Schwartz and Cohen report that feedback is most effective in changing behavior when it is delivered in a timely fashion, is combined with both education and either incentives or administrative changes (e.g., the reorganization of charts in a computerized or problem-based format), and includes comparisons with other peers. In practical terms, one way to offer feedback is to audit the medical records for a group of physicians and provide each physician with an individual performance rating relative to his or her peers. The physician could receive this feedback in a confidential report, perhaps as part of an educational session on alcohol screening. In addition, showing a slide during the session that anonymously lists each physician’s rating can be a powerful motivation for change.

Other studies suggest additional approaches to providing feedback. Morrow and colleagues (1995) describe a framework for changing preventive health care behavior by combining peer review, feedback, and financial incentives. Payne and colleagues (1984) demonstrated an improvement in outpatient care following feedback to a group of physicians attending a seminar in which they participated in problem identification, problem-solving, and solution implementation. The resulting improvement was reinforced through followup consultations after the seminar.

A report by Ockene and colleagues (1997) provides some of the first data on the use of feedback in the alcohol field. This study trained 31 clinicians (i.e., faculty, residents, and advanced nurse practitioners) in techniques for providing brief advice counseling to patients for alcohol use disorders. Each clinician participated in a 90-minute training workshop followed by a 30-minute one-on-one feedback session 2 to 6 weeks later. Standardized patients were used to rate the clinical skills of the participants before and after the workshop and feedback sessions. A comparison of the “before” and “after” ratings demonstrated significant improvements in the clinicians’ skills, attitudes, and knowledge related to alcohol and alcohol screening.

As suggested in this brief review of the literature, the provision of feedback can change physician behavior and clinical practice. Eisenberg and Williams (1981) suggest that feedback works by capitalizing on the health care provider’s sense of achievement and desire to excel. Regardless of the reason why feedback works, however, the success of this strategy makes it appealing for application in the alcohol field.

### Academic Detailing

Academic detailing refers to clinic-based educational activities focused on individual practitioners. These educational activities involve outreach visits to offer short didactic presentations to physicians, skills training through role playing, performance feedback, or discussions on pertinent topics (e.g., how to overcome staff resistance to incorporating new procedures). Studies have focused on a variety of health care professionals, such as physicians, nurses, pharmacists, and health educators, to conduct these office-based outreach visits. In addition, pharmaceutical companies have employed this strategy effectively to encourage physicians to prescribe certain medications.

Soumerai and Avorn (1990) examined face-to-face outreach visits by clinical pharmacists and the provision of written materials and compared the effects that these two methods had on changing physicians’ prescribing patterns. In this study, 435 physicians were assigned randomly to receive one of the two experimental methods and were assessed for changes in their prescribing patterns. The results showed that educational visits significantly changed the physicians’ prescribing patterns. In addition, the strength of the effects depended on the number of one-on-one followup visits by the clinical pharmacist: the more visits, the greater the change in prescribing patterns. The study concluded that brevity, repetition, and reinforcement of recommended practices are important elements in changing physician behavior.

### Financial Incentives or Penalties

Research suggests that financial incentives are another effective tool for changing clinician behavior. Incentives can be based on a variety of indicators, such as the number of patients immunized, the frequency of screening for a selected health problem (e.g., mammography for women over age 50), the number of prescriptions written for selected medications (e.g., expensive antibiotics), the number of patients referred to specialty care, or the number of patients hospitalized. Positive incentives can include bonuses, higher base salaries, or increases in the negotiated rate a managed care organization pays physicians per enrolled patient (i.e., capitation payments). These types of incentives can be powerful motivators. For example, Hickson and colleagues (1987) performed a randomized clinical trial to determine whether pediatric residents who were paid per patient would attend more patients in the clinic (and thereby become more efficient in preparation for their future work) compared with residents who received a fixed salary. Not surprisingly, the residents who were paid per patient took care of significantly more patients, implying that the financial incentive was an effective motivator.

Negative financial incentives (i.e., penalties) also produce changes in behavior, as found in another study by Hillman and colleagues (1989). This study examined rates of patient hospitalization among a group of primary-care physicians who were at personal financial risk for referral and hospital care. The results indicated that the rate of patient hospitalization decreased after this reimbursement policy was implemented.

Although additional research in this area is warranted, one can reasonably assume that creating financial incentives for physicians could be applied to the alcohol field to facilitate the implementation of alcohol-screening procedures. As an example, managed care companies could review medical records and provide a year-end bonus to physicians who screened a predetermined percentage of patients for alcohol problems in the preceding year.

## Implementation of Alcohol Screening

To incorporate routine alcohol screening efficiently, physicians can adapt a comprehensive clinic-based program similar to the programs used to screen for other health concerns, such as high blood pressure, cancer, elevated cholesterol levels, and tobacco use. In many clinical practices, screening for these health concerns already has become a routine element of care and usually includes procedures such as patient questionnaires, physical measurements or laboratory tests, manual or computerized reminder systems to ensure a thorough examination and assist with followup, standardized prevention messages, and protocol-driven treatment methods.

Clinic-based systems acknowledge the complexity of implementing a new activity into a busy practice and the need to systematize the activity as part of routine care. In addition, a clinic-based system requires the active participation of all staff members, not just the individual clinician responsible for questioning the patients. Front-desk staff, for example, often distribute questionnaires and attach reminder printouts to patient charts. Nurses score the questionnaires and follow established protocols designed to manage positive and negative responses. Medical record clerks record the information in the charts and in databases. Physicians then use the data for clinical decisionmaking.

The effectiveness of clinic-based systems has been an active area of research since the early 1980’s (Kottke et al. 1988). For example, Solberg and colleagues (1990) conducted a study on a clinic-based system designed to establish a smoking cessation program. The program included patient interviews to screen for current smoking status, chart labels (i.e., color-coded stickers placed on the outside of the chart to indicate the patient’s smoking status), brief messages advising patients of the importance of smoking cessation, reminder cards attached to the patient’s medical record to prompt physicians to inquire about smoking status during the visit, and followup telephone calls by clinic nurses. After 1 year, the researchers reported smoking cessation rates of greater than 20 percent.

Black and colleagues (1995) reported on a study designed to assess the effect of preprinted, structured, complaint-specific patient encounter forms (i.e., “quick sheets”) on the documentation, resource use, and treatment of emergency room patients. These quick sheets aimed to guide care for common clinical conditions (e.g., asthma, sore throat, and cuts) and were based on expectations developed by medical staff in the emergency medicine field. Study results demonstrated a variety of positive outcomes, including improved documentation of the patient’s history and physical findings, decreased use of clinical tests and medications, and decreased costs.

For alcohol screening, a comprehensive clinic-based program could include the following components:

- Questionnaires administered to patients by the receptionist or nurse, preferably with the alcohol questions embedded among general health questions
- A readily available assessment tool, such as one of the instruments discussed in the next section
- A computerized reminder system maintained by clerical staff to prompt clinicians to screen patients for alcohol use disorders or to follow up on previous treatment recommendations
- A list, which is periodically updated by clerical staff, of alcohol specialists, peer-support meetings (e.g., Alcoholics Anonymous or Al-Anon), and community support agencies.

Although the ultimate goal is to provide alcohol screening for all patients, screening in clinical settings could initially focus on particular high-risk groups, such as patients who are pregnant, suffering traumatic injuries, or receiving medication for high blood pressure or depression. Alcohol screening also could serve as one component of several targeted health issues, such as breast cancer or tobacco use screening.

### Choosing a Screening Instrument

Several alcohol screening instruments with good accuracy are available for use in health care settings, including the instruments discussed here and in the related article by Cherpitel (see pp. 348–351). Each screening instrument has particular strengths and weaknesses and varies in its applicability to clinical settings. When selecting a screening procedure for routine implementation, clinicians and health care systems should consider factors such as the goals of the screening process, the target population (e.g., young adults, pregnant women, or the elderly), intervention options, clinician training needs, and costs.

The physicians’ guide developed by NIAAA recommends quantity/frequency and binge-drinking questions (see text box, p. 348) as the primary screening test (NIAAA 1995). These questions are sensitive (i.e., they correctly identify patients with alcohol use disorders in a high percentage of cases) and have a low rate of false-positive results. The questions are easy to use and can be incorporated into a physician’s practice with minimal cost and effort. Although patients sometimes underreport their alcohol use—particularly patients who are alcohol dependent or intoxicated—underreporting can be minimized with the use of appropriate interview techniques (i.e., a direct, nonjudgmental approach); collaborative reports (i.e., family member reports and medical record reviews); and laboratory tests (i.e., breath analysis; blood alcohol level; or levels of other excessive alcohol consumption indicators, such as the enzyme gamma-glutamyl transferase or the blood component carbohydrate-deficient transferrin).

When screening patients for current or lifetime alcohol dependence, NIAAA’s physicians’ guide recommends using the CAGE questions (see CAGE text box, p. 349). As many as 50 percent of at-risk drinkers will not be identified if questions are limited to the four components of the CAGE test, however (Adams et al. 1996). To avoid missing the identification of at-risk drinkers, clinicians can use general health-screening questionnaires that include the CAGE questions, such as the PRIME-MD (Spitzer et al. 1994) or the Health Screening Survey (Fleming and Barry 1991).

Some alcohol-screening instruments work best with specific patient populations. Health care systems that primarily focus on women, for example, may want to use the TWEAK test (see TWEAK text box, p. 349) or a similar instrument, such as T-ACE, that has been designed and tested specifically for use with women. Emergency-care clinicians should consider using the Rapid Alcohol Problems Screen (RAPS), which appears to have advantages over other screening methods when applied in emergency-care settings.

Regardless of which alcohol screening instrument is selected, clinicians also may want to establish a brief assessment procedure for patients who screen positive. Examples of pencil-and-paper assessment tools for use in general health care settings include the 10-question Alcohol Use Disorders Identification Test, the 25-question Michigan Alcohol Screening Test, and the 15-question Short Alcohol Dependence Data Questionnaire (Davidson and Raistrick 1986). Clinicians should use an assessment procedure to determine where a patient is on the spectrum of alcohol use (i.e., whether the patient is a low-risk, at-risk, problem, or dependent drinker) before proceeding with a therapeutic plan, which may range from brief intervention to referral to an alcohol treatment program.

A positive alcohol screen can have enormous implications for a patient, possibly affecting his or her employment status, ability to obtain insurance, and status in the community. Therefore, clinicians should diagnose an alcohol use disorder with the same caution used to diagnose other medical problems.

## Summary

The U.S. health care system provides a great opportunity to identify the majority of people adversely affected by alcohol use disorders. Several specific and sensitive screening tests are available to help clinicians implement routine alcohol screening in their practices. In addition, brief intervention trials have found that simply asking questions about alcohol use can reduce levels of drinking (Bien et al. 1993). The challenge, however, is to incorporate alcohol-screening procedures in the context of a multitude of other clinical activities and screening programs. For example, alcohol screening must compete and fit in with screening for immunization status, breast cancer, colon cancer, prostate cancer, cholesterol level, and smoking status, all of which have become high priorities in managed care systems.

The incorporation of routine screening and treatment for high blood pressure and high cholesterol levels in the 1980’s did not occur in the U.S. health care system until research demonstrated that screening for these conditions reduced the frequency of illnesses and deaths (Veterans Administration Cooperative Study Group on Antihypertensive Agents 1967; Multiple Risk Factor Intervention Trial Research Group 1982). Although decreased health care utilization and costs were not a major factor in the acceptance of routine screening for high blood pressure and cholesterol levels as the “standard of care,” the emergence of managed care systems demands that any new screening procedures be cost-effective. Therefore, additional research is needed to establish that alcohol screening, brief intervention, and referral to specialized alcohol treatment care truly reduce disease, deaths, health care utilization, and costs. In particular, limited information currently exists on the cost-effectiveness of alcohol screening and brief intervention (French in press).

Changing physician behavior is a complex endeavor not to be taken lightly. The process of change is similar to changing a patient’s behavior regarding alcohol use—knowledge and education are not enough. Educational endeavors must be expanded in medical schools, residency training sites, and CME programs to include role playing and skills-based workshops on alcohol screening. In addition, new physicians should be tested on their alcohol-screening skills as part of the requirement for graduation from medical school and residency. Standards of care also must change so that all patients admitted to hospitals and seen in outpatient clinics are screened for alcohol use disorders just as they are screened for high blood pressure, tobacco use, and high cholesterol levels. Accreditation groups (e.g., the Joint Commission, which accredits hospitals; the American Association of Medical Colleges, which accredits medical schools; and the 24 Residency Review Committees, which accredit residency programs) have the opportunity to require routine alcohol screening and adequate training for students and residents.

Although alcohol screening for all patients is not yet the current standard of care, the acceptance of routine screening nevertheless has come a long way over the last 20 years, as the following examples demonstrate:

- Most medical schools and residency programs now provide educational programs on alcohol screening.
- Many hospitals are developing alcohol consulting services.
- Most health forms given to new patients in hospitals and outpatient clinics, as well as many nursing intake forms, now include alcohol-screening questions.
- Screening all pregnant women for alcohol use is becoming the standard of care in many areas of the country.
- Some managed care companies are beginning to include (i.e., “carve in”) specialized alcohol treatment services under primary care in order to facilitate referral and communication.
- More than a dozen brief intervention clinical trials currently being supported by NIAAA should provide evidence to convince the managed care industry that alcohol screening and brief intervention are cost-effective.
- The number of medical school faculty who work in the alcohol area continues to increase.

By applying the knowledge and experience gained in changing physician behavior and systems of care in other areas of medicine, the goal of routine alcohol screening for all patients in the U.S. health care system appears to be within reach.

## Footnotes

1 A standard drink is defined as one 12-ounce bottle of beer, one 5-ounce glass of wine, or 1.5 ounces of distilled spirits.
